# Evolutionary Significance of NHX Family and NHX1 in Salinity Stress Adaptation in the Genus *Oryza*

**DOI:** 10.3390/ijms23042092

**Published:** 2022-02-14

**Authors:** Celymar Angela Solis, Miing-Tiem Yong, Meixue Zhou, Gayatri Venkataraman, Lana Shabala, Paul Holford, Sergey Shabala, Zhong-Hua Chen

**Affiliations:** 1 School of Science, Western Sydney University, Penrith, NSW 2751, Australia; c.solis@westernsydney.edu.au (C.A.S.); m.yong@westernsydney.edu.au (M.-T.Y.); p.holford@westernsydney.edu.au (P.H.); 2 Tasmanian Institute of Agriculture, University of Tasmania, Hobart, TAS 7001, Australia; meixue.zhou@utas.edu.au (M.Z.); l.shabala@utas.edu.au (L.S.); 3 Plant Molecular Biology Laboratory, M. S. Swaminathan Research Foundation, III Cross Street, Taramani Institutional Area, Chennai 600113, India; gayatri@mssrf.res.in; 4 International Research Centre for Environmental Membrane Biology, Foshan University, Foshan 528000, China; 5 Hawkesbury Institute for the Environment, Western Sydney University, Penrith, NSW 2751, Australia

**Keywords:** gene family evolution, wild rice, sodium homeostasis, *Oryza sativa*, *Oryza coarctata*

## Abstract

Rice (*Oryza sativa*), a staple crop for a substantial part of the world’s population, is highly sensitive to soil salinity; however, some wild *Oryza* relatives can survive in highly saline environments. Sodium/hydrogen antiporter (NHX) family members contribute to Na^+^ homeostasis in plants and play a major role in conferring salinity tolerance. In this study, we analyzed the evolution of NHX family members using phylogeny, conserved domains, tertiary structures, expression patterns, and physiology of cultivated and wild *Oryza* species to decipher the role of NHXs in salt tolerance in *Oryza*. Phylogenetic analysis showed that the NHX family can be classified into three subfamilies directly related to their subcellular localization: endomembrane, plasma membrane, and tonoplast (vacuolar subfamily, *vNHX1*). Phylogenetic and structural analysis showed that *vNHX1s* have evolved from streptophyte algae (e.g., *Klebsormidium nitens*) and are abundant and highly conserved in all major land plant lineages, including *Oryza*. Moreover, we showed that tissue tolerance is a crucial trait conferring tolerance to salinity in wild rice species. Higher Na^+^ accumulation and reduced Na^+^ effluxes in leaf mesophyll were observed in the salt-tolerant wild rice species *O. alta*, *O. latifolia*, and *O. coarctata*. Among the key genes affecting tissue tolerance, expression of *NHX1* and *SOS1/NHX7* exhibited significant correlation with salt tolerance among the rice species and cultivars. This study provides insights into the evolutionary origin of plant NHXs and their role in tissue tolerance of *Oryza* species and facilitates the inclusion of this trait during the development of salinity-tolerant rice cultivars.

## 1. Introduction

Soil salinity causes major issues for world food production [1], and areas with saline soils are predicted to expand due to low precipitation, elevated surface evaporation, irrigation with saline water, and poor agricultural practices [2]. Salinity inhibits plant growth and reduces both the yield and quality of crops [3,4]. Among the cereals, rice (*Oryza sativa* L.) is a staple food of two-thirds of the global population. At a threshold soil electrical conductivity (EC) of ~3 dS m^−1^, salinity stress can result in significant growth retardation and yield loss of this crop [5], and on average, a 12% yield loss occurs with every dS m^–1^ rise in soil EC. A 50% yield loss was recorded in soil with an EC of ~6 dS m^−1^ for most high-yielding varieties [6]. Salinity stress mainly affects plants in three ways: by causing osmotic stress, thus restricting water uptake; by imposing ionic stress from increased uptake of toxic Na^+^ and Cl^−^ ions causing ionic imbalances; and by causing oxidative damage that leads to reduced plant growth and senescence [7,8]. Plants have evolved a series of adaptive strategies to regulate various physiological, biochemical, and molecular responses to cope with salinity [9]. These responses to salinity are not universal, and effects vary with growth stage, severity of the stress, duration, and tolerance of a species and genotypes within a species [10].

Similar to other cereal crops, rice genotypes are relatively tolerant at the germination stage but highly susceptible at the early seedling stage (1–3 weeks) and then become slightly more tolerant from active tillering to the reproductive stage. The most susceptible stage as far as overall yield losses are concerned is at panicle initiation to flowering and early grain filling [5,11]. Despite major efforts, only a small number of salinity-tolerant rice accessions have been identified so far [12,13,14]. Domesticated rice has been selected mainly for its high yield, resulting in low variation in salinity tolerance within the species [15]. One solution to overcome the lack of suitable donors with salinity tolerance is the use of wild rice relatives. Wild relatives of domesticated crops have been used for crop improvement [16] and are often capable of surviving unfavorable conditions, such as salinity, due to the presence of traits that have been lost during the course of the domestication process [17]. One example is halophytic wild rice *O. coarctata* Roxb., a species that can survive saline conditions up to 40 dS m^−1^; this wild rice species is commonly found growing as a mangrove associate in tidal and semi-tidal inundation areas [18]. Identifying and harnessing the key genes responsible for such high salinity tolerance in crop wild relatives and understanding how the trait has evolved will help identify the possible donors available for improvement [19]. However, the mechanisms associated with salinity tolerance in wild rice remain poorly understood, as most studies have focused on domesticated varieties, which have limited genetic diversity with respect to salinity tolerance [20].

One of the key salinity tolerance mechanisms that has been identified in wild rice is tissue tolerance [13,21,22,23,24]. Tissue tolerance is the ability of the plant to tolerate high levels of NaCl in its tissues while retaining chlorophyll, maintaining leaf water potentials and photosynthetic activity, as well as other vital cellular functions. This type of tolerance can be achieved by sequestering excess Na^+^ into the vacuole, thereby avoiding a build-up of toxic levels of this ion in the cytoplasm [8,25,26]. This process is called vacuolar Na^+^ sequestration, and is considered to be a key trait in halophytes such as the monocot *Oryza coarctata* [27,28,29,30] and the eudicot *Salicornia europaea* [31,32,33]. Sequestered Na^+^ ions act as a cheap osmoticum for maintaining cell turgor pressure and allow shoot expansion and growth to occur under saline conditions [34,35]. This process is made possible by the operation of tonoplast Na^+^/H^+^ antiporters (NHXs). Na^+^/H^+^ antiporters exchange protons for Na^+^ ions across membranes and are particularly active in the vacuoles of plants, algae, and fungi [27,28,29,30]. NHXs remove Na^+^ from the cytosol by pumping H^+^ into the vacuole using two proton pumps: vacuolar H^+^-inorganic pyrophosphatase (V-inorganic pyrophosphatase [V-PPase], E.C. 3.6.1.1) and vacuolar H^+^-ATPase (V-ATPase, E.C. 3.6.1.3) [36]. Extensive studies on Arabidopsis and rice have demonstrated the key roles NHX plays in salinity tolerance in which plants overexpressing *NHXs* have the ability to retain K^+^ ions in the cytosol under saline conditions, thereby increasing tolerance [36,37,38,39]. However, the molecular mechanisms and evolutionary origin of this transporter with respect to salinity tolerance [40,41,42] are yet to be examined in wild *Oryza* species.

In this study, we hypothesized that the evolution and diversification of NHX1 in the *Oryza* genus contributes to distinct levels of salinity tolerance among wild and cultivated rice species. To understand the evolutionary origin of this aspect of salinity tolerance of wild *Oryza* species, we used a combination of physiological, molecular, and evolutionary analyses to compare the responses to salinity of wild and domesticated accessions. We also provide evidence of a significant contribution of tissue tolerance in differential salinity tolerance found between wild and cultivated rice. Comprehensive evolutionary and molecular analyses shed light on the positive role of NHX1 in the adaptation of rice to salinity stress. We also identified accessions of wild *Oryza* species that could be promising sources of tissue tolerance traits. Taken together, this study will be useful to promote the use of wild *Oryza* genetic resources for identification of candidate genes and crop improvement.

## 2. Results

### 2.1. Evolution of NHX Genes in Green Plants

**Figure 1:**
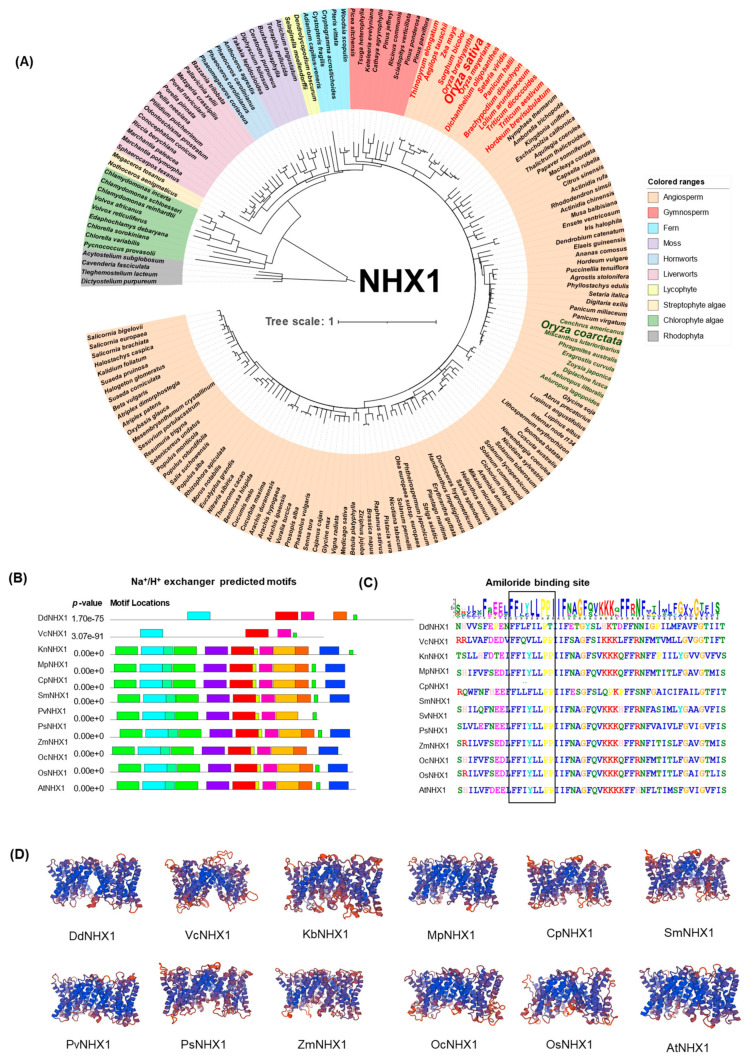
Phylogenetic and structural analysis of NHX1 in land plants and algae. (**A**) Phylogenetic relationship of NHX1 proteins in representative species of major lineages of plants and algae. The tree was constructed using maximum likelihood with 1 × 10³ bootstrap replicates. (**B**) Analysis of the predicted motifs of the NHX1. (**C**) Sequences of amiloride binding site motif. (**D**) Predicted 3D structure of NHX1 proteins with conserved alpha helices highlighted in blue and random coils in red. Dd, *Dictyostelium discoideum* (outgroup); Vc, *Volvox carteri*; Kr, *Klebsormidium nitens*; Mp, *Marchantia polymorpha*; Cp, *Ceratodon purpureus*; Sm, *Selaginella moellendorffii*; Pv, *Pteris vittata*; Ps, *Picea sitchensis*; Zm, *Zea mays*; Oc, *Oryza coarctata*; Os, *Oryza sativa*; and At, *Arabidopsis thaliana*.

To trace the evolution of the NHXs responsible for vacuolar compartmentalization of Na^+^ ions in diverse plant species, we used the *Arabidopsis thaliana* Na^+^/H^+^ antiporter gene, *AtNHX1*, the first cloned NHX homologue [43], as a query to perform BLASTp searches from the One Thousand Plant Transcriptome (OneKP) and Phytozome genome databases. We surveyed and selected representative species that are phylogenetically important in land plant evolution [44,45]. The predicted motifs and phylogeny of NHXs from species selected from OneKP showed that NHX1 is found among diverse green plant species, with a likely origin from streptophyte algae (e.g., *Klebsormidium nitens*) (Figure 1A,C). NHX proteins in most of the species contain a conserved amiloride binding site (FFI/LY/FLLPPI) in Motif 1 of the gene in rhodophyte and chlorophyte algae and in Motif 2 of the gene in streptophyte algae and embryophytes (Figure 1C). The number of deduced amino acids varies from 463 to 674 with variable sequence homology in the tested representative species of the major lineage of green plants (Figure S1, Table S1). Interestingly, we found that eudicots and monocots tend to have higher numbers of orthologues of vacuolar NHXs (vNHXs) than those in early divergent green plant species (Table S2). In addition, *OsNHX1* from cultivated rice showed a closer phylogenetic relationship to those from other glycophytic members of the Poaceae, e.g., *Zea mays*, *Sorghum bicolor*, and *Hordeum vulgare* (red font), whereas *OcNHX1* from the halophilic wild rice, *Oryza coarctata*, showed a closer relationship with known halophilic grasses, such as *Zoysia japonica*, *Diplachne fusca*, and *Aeluropus lagopoides* (green font) (Figure 1A). The predicted 3D structural models of the NHX1s of the representative species were constructed using the QMEANDisCo rule [46], with all models containing highly conserved alpha helices (highlighted in blue) followed by random coils (Figure 1D).

### 2.2. Phylogeny, Classification, and Molecular Characterisation of NHXs in Rice

**Figure 2:**
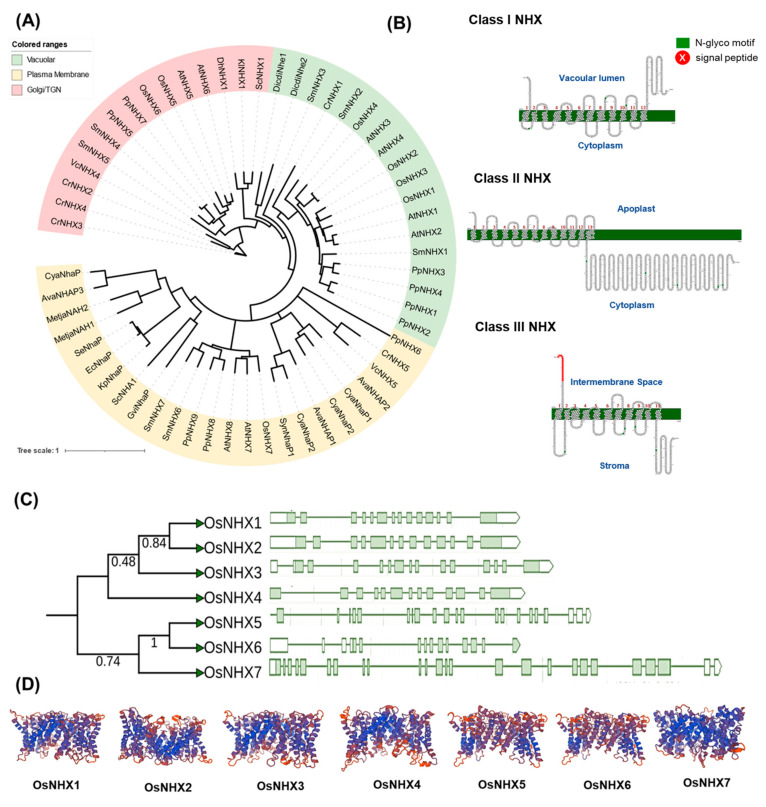
Phylogenetic and structural analysis of NHX transporters in *Oryza* species. (**A**) Phylogenetic relationship of three major classes of NHX proteins (vacuolar, endosome, and plasma membrane) marked with different colors in representative species of major lineages of plants and algae. The phylogenetic tree was constructed using maximum likelihood with 1000 bootstrap replicates. (**B**) Topology models of three major classes of NHX proteins (labeled as vacuolar, plasma membrane, or trans-golgi network). (**C**) Sequence phylogeny and gene characterization of NHX transporters in *Oryza sativa*. Exons are represented as green boxes and introns as green lines. (**D**) Predicted 3D structure of NHX proteins in rice with conserved alpha helices highlighted in blue and random coils in red.

Seven putative *OsNHX* genes (*OsNHX1–7*) were identified in the *Oryza sativa* genome. The number of amino acids deduced from these genes ranges from 462–1149 with corresponding molecular masses of 31–128 kDa and theoretical isoelectric points (pIs) varying from 5.53 to 8.34 (Table S3). To elucidate the evolutionary and phylogenetic relationships among the NHX proteins in *Oryza* species and representative species from major plant lineages, an unrooted phylogenetic tree was constructed. We found that the NHX gene family is grouped into three subfamilies/classes based on their subcellular localization consistent with previous studies in *Arabidopsis*: Class I (Vac-class), Class II (Endo-class), and Class III (PM-class) (Figure 2A,B). Topological prediction of the *OsNHXs* suggests 12, 13, and 11 transmembrane (TM) domains for Classes I, II, and III, respectively (Figure 2B). Interestingly, genome browsing showed that Vac-class NHXs are the most abundant in rice, with four homologues being identified. In addition, the number of putative NHXs mapped among the diploid *Oryza* species were highly conserved, with similar numbers of NHX homologues mapped per subclass in the representative *Oryza* species (Table S4). Analysis of gene structure examining exon-intron organization shows Vac-class NHXs (*OsNHX1–4*) had 13–14 introns, Endo-class NHXs (*OsNHX5–6*) had 13–18 introns, and PM-class NHXs (*OsNHX7*) had 23 introns (Figure 2C). The intron number, exon length, and intron phase were relatively conserved among the members of the same clade. In addition, the evolutionary conservation of nucleotide sequences among Vac-NHXs in *Oryza* was also supported by amino acid sequence identity. Vac-NHX homologs exhibited high sequence identities at the amino acid level (63–82%), while other rice NHX subfamilies showed lower identities (50%) (Figure S2). Mapping of the protein sequences of *OsNHX1–7* to the genomes of representative species from the major plant lineages confirmed their functional classifications based on subcellular localization (Figure 2A).

**Figure 3:**
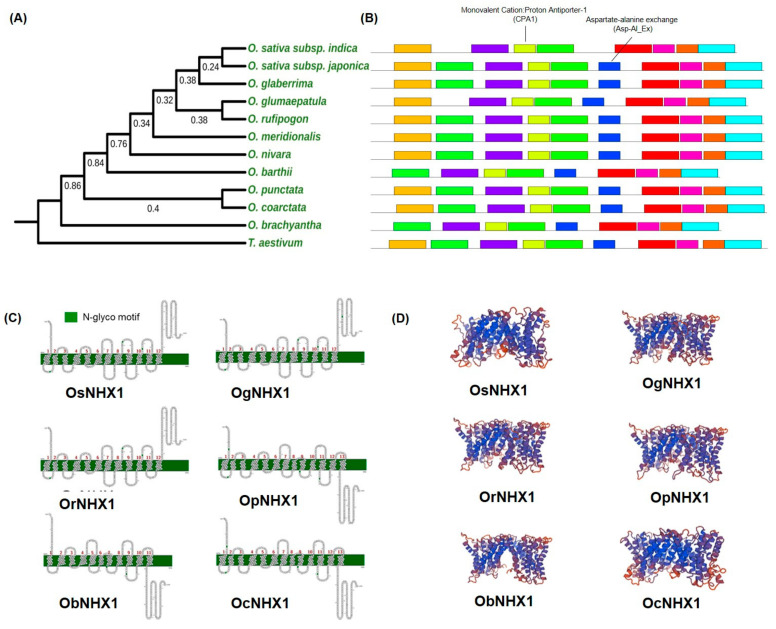
Gene structure and conserved sequences of NHX1 in *Oryza* species. (**A**) Phylogenetic relationships of NHX1s. The tree was constructed using *Oryza* NHX1 protein sequences using the neighbor-joining method in MEGA X. The bootstrap consensus tree was inferred from 1000 replicates. (**B**) Sequence logo of the most variable domain mapped in NHX1. Motif analysis was performed using MEME 5.4 software. Different motifs, numbered 1–10, are displayed as different colored boxes. The names of the motifs are presented in Supplementary Figure S4. (**C**) Transmembrane domains of VvNHX proteins constructed with Protter. (**D**) Ribbon representation of the predicted models of the *Oryza* NHX1 exchangers with conserved alpha helices highlighted in blue and random coils in red. Os, *Oryza sativa*; Oc, *Oryza coarcta*; Ob, *Oryza brachyantha*; Op, *Oryza punctata*, Or, *Oryza rufipogon*; Og, *Oryza glaberrima*.

We also examined the structural diversity and structural characteristics of v*NHX1* genes within the genus *Oryza* in relation to their possible contributions to salinity tolerance in wild and cultivated rice. For this, a phylogenetic tree was constructed with putative NHX1 homologs obtained from publicly available *Oryza* genomes. The resulting phylogeny showed that NHX1 is highly conserved in *Oryza* species with 93.9% overall pairwise identity (Figure 3A and Figure S3). The number of deduced amino acids varies from 475–539, with an average molecular weight of 57.44 kDa (Table S5). MEME results show the conserved NHX1 domains in the 11 *Oryza* species that were examined (Figure 3B). Overall, the predicted motifs of *Oryza* NHX1s range from 29–50 amino acid residues (Figure S4) and have 11–13 predicted transmembrane regions (Figure 3D). Interestingly, NHX1s of two salt-tolerant wild rice species, including *O. coarctata*, were predicted to have 13 transmembrane domains. Moreover, all representative *Oryza* species have a conserved amiloride binding domain (FFYLFL) in the 3rd and 4th motifs, predicted functional monovalent cation/proton antiporter (CPA1) family conserved domains of NHX1 proteins (yellow), and FLFLYV in the 5th or 6th motifs (blue) with predicted functional aspartate-alanine exchanger (Figure 3B and Figure S4). The predicted 3D structure of the representative *Oryza* NHX proteins showed that at least 50–60% of the amino acid sequences had been modeled with high confidence (Figure 3D).

### 2.3. Physiological Evidence for Diversity of Tissue Tolerance in Oryza Species

**Figure 4:**
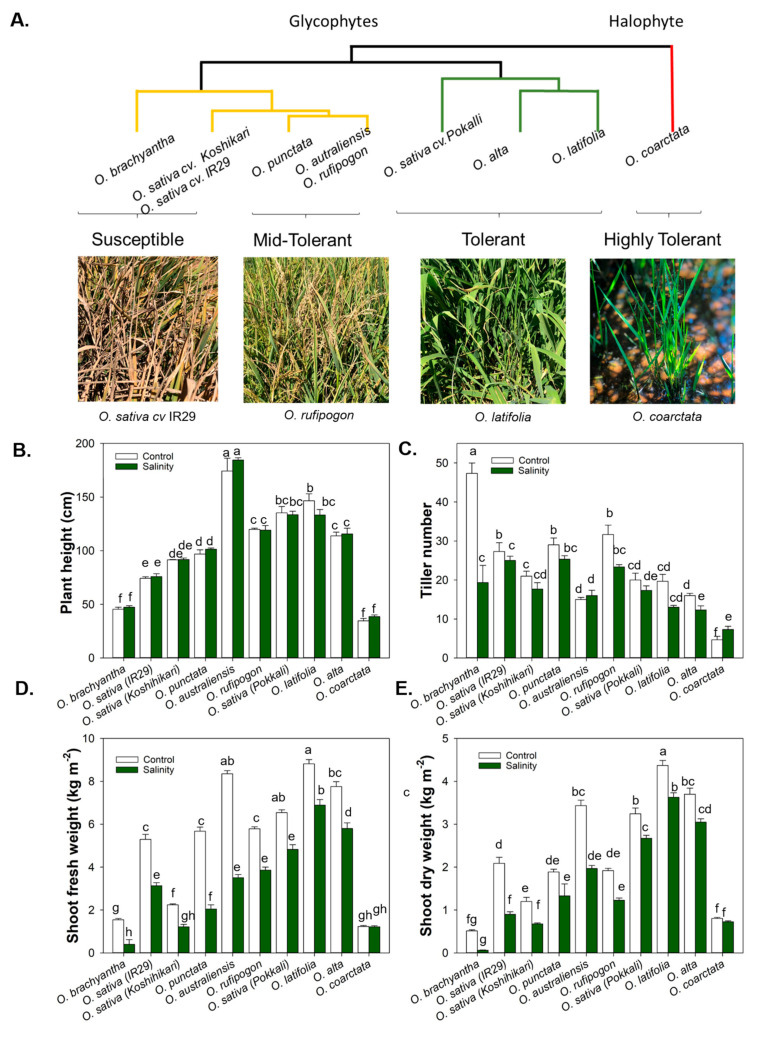
Variation of salinity tolerance in *Oryza* species. Phylogenetic relationships based on a Euclidian matrix on responses of members of the genus *Oryza* to salinity stress and morphological variation under control and salinity stress conditions. (**A**) Phenotypic variation in the effect of salinity treatment on overall growth, (**B**) plant height, (**C**) tiller number, (**D**) fresh weight, and (**E**) dry weight. Different lowercase letters indicate significant differences at *p* < 0.05 (n = 3–6 *Oryza* lines) according to Duncan’s Multiple Range Test.

In this study, cultivated and wild rice species exhibited a large difference in salinity tolerance when subjected to long-term salinity stress of 12 dS m^–1^ at the vegetative stage. Phenotypic data were analysed using a hierarchical clustering approach based on all the morphophysiological traits measured four weeks after salinity stress imposition (Figure 4A). For susceptible *Oryza* genotypes, symptoms were observed as early as one week after salinity stress imposition. The visual symptoms observed included leaf necrosis at the tips of the leaves, leaf yellowing, leaf rolling, and eventually leaf and tiller senescence, leading to reduced photosynthesis and yield loss (Figure 4). The symptoms were more severe in *Oryza* species *O. brachyantha*, *O. sativa* cv. IR29, and *O. sativa* cv. Koshihikari. The susceptible genotypes also had significant reductions (ANOVA species × treatment effects, *p* < 0.05) in tiller number (Figure 4C) and shoot biomass (Figure 4D,E). No significant difference in plant height was found between the control and salinity-stressed plants (Figure 4B, ANOVA treatment effect, *p* > 0.05). In comparison, the tolerant cultivated rice *O. sativa cv.* Pokkali is close to salinity tolerance exhibited by wild rice species *O. latifolia, O. alta*, and *O. coarctata*. (Figure 4A–E) with respect to relative plant height, tiller number, and fresh weight (Figure S5).

**Figure 5:**
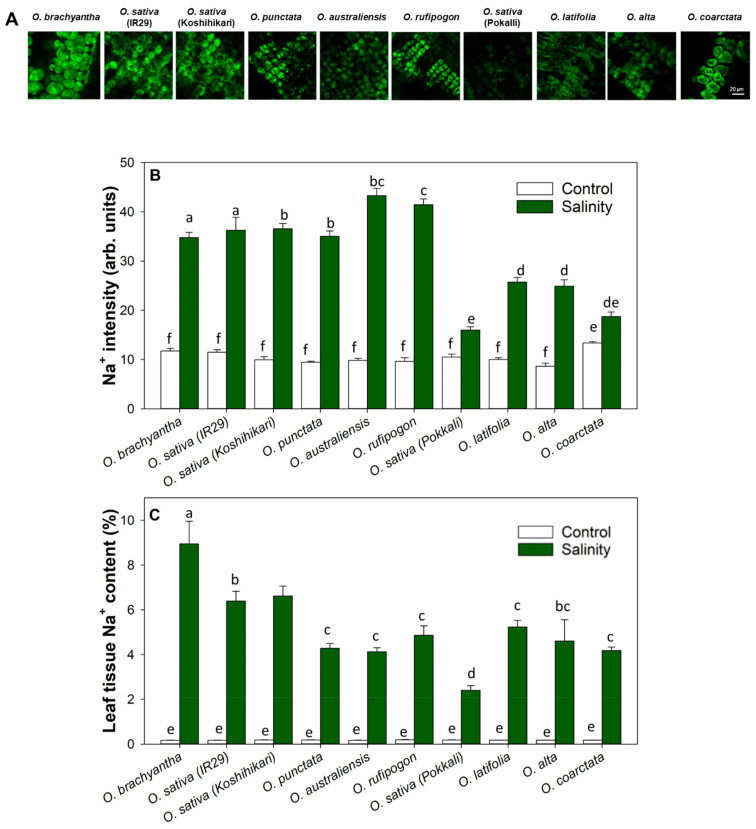
Na^+^ accumulation in leaf mesophyll cells under salinity treatment in *Oryza* species. (**A**) Representative images of fluorescence due to Na^+^ in mesophyll after 4 weeks of salinity stress. The scale bar = 20 µm. (**B**) Relative mean Na^+^ intensity in mesophyll cells and (**C**) leaf Na^+^ content of control and salinity stress rice. Lowercase letters indicate significant differences at *p* < 0.05 (n = 6 biological replicates) according to Duncan’s Multiple Range Test.

To examine the tissue tolerance among *Oryza* species, the accumulation of Na^+^ ions in leaf mesophyll cells was measured using the Na^+^-specific fluorescence of peeled leaf tissues that had exposed photosynthetically active mesophyll cells. The green fluorescence signal is directly proportional to the sodium content in the samples. The salinity treatment significantly increased Na^+^ contents in the mesophyll cells of all lines, but more accumulation was evident in the susceptible lines in which an approximately 4-fold higher fluorescence intensity was observed. This accumulation in mesophyll cells translated to overall Na^+^ content in the leaf tissue upon harvest at complete maturity. (Figure 5B) Among the salinity tolerant lines, based on the salinity tolerance scores (Table S6), salt-tolerant landrace Pokkali showed the lowest accumulation of Na^+^ in the leaf, whereas the salt-tolerant wild species (*O. latifolia, O. alta*, and *O. coarctata*) accumulated significantly more Na^+^ ions (Figure 5A) without affecting their physiological performance under salinity stress at the vegetative stage (Figure 4).

### 2.4. Linking Genetic Diversity of NHXs and Transporter Genes with Tissue Tolerance to Salinity in Oryza

**Figure 6:**
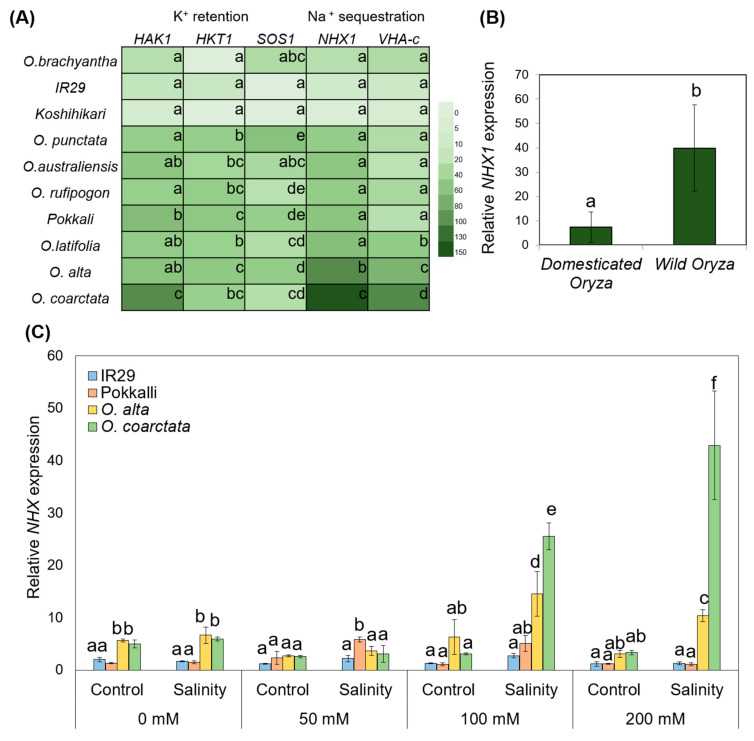
Gene expression and tissue tolerance in *Oryza* species under salinity treatment. (**A**) Differential expression profiling of known genes for K^+^ retention (*HAK1*, *HKT1*, and *SOS1/NHX7*) and vacuolar sequestration (*NHX1 AND VHA)* of the 7 wild rice and 3 cultivated rice cultivars used in this study. Lowercase letters indicate significant differences at *p* < 0.05 (n = 6 biological replicates) according to Duncan’s Multiple Range Test per gene. (**B**) A comparison of the expression of NHX1s in domesticated (IR29, Koshihikari, Pokkali) and wild rice species (*O. brachyantha*, *O. punctata*, *O. rupifogon*, *O. autraliensis*, *O. alta*, *O. latifolia*, *O. coarctata*). (**C**) Differential expression of *NHX1* in representative susceptible (IR29) and tolerant (Pokkalli, *O. alta*, *O. coarctata*) *Oryza* species at increasing concentrations of salinity. Lowercase letters indicate significant differences at *p* < 0.05 (n = 6 biological replicates) according to Duncan’s Multiple Range Test.

To further confirm the evidence for differential Na^+^ accumulation in the leaves, we performed quantitative real-time PCR (qPCR) of genes known to be associated with salinity tolerance through K^+^ retention [high affinity potassium transporter 1 (*HAK1*), high affinity potassium transporter 1;4 (*HKT1*), Na^+^/H^+^ antiporter (*SOS1/NHX7*)] and vacuolar Na^+^ sequestration (*NHX1* and *VHA-c*). A significant upregulation of key transporters was observed in the salt-tolerant cultivated and wild rice species in response to salt stress. One of the most interesting findings was that the expression of *HAK1*, *VHA-c*, and *NHX1* was highest in the halophytic wild rice, *Oryza coacrctata*, with 77-, 80-, and 130-fold NaCl-induced increases in expression, respectively (Figure 6A). The expression of genes affecting K^+^ retention were significantly higher in Pokkali compared to tolerant wild rice species, *O. alta* and *O. latifolia*, with 30-, 17-, and 10-fold increases in expression of *HAK1*, *HKT1*, and *SOS1*, respectively, which is almost double the expression of these genes compared to those in salt-tolerant wild rice *O. latfolia.* Strikingly, *NHX1* was upregulated in the susceptible line *O. brachyanta* under salinity stress (Figure 6A,B). We also found that the transcript abundance of *NHX1* varies in a salinity dose-dependent manner in wild *Oryza* species (Figure 6C).

**Figure 7:**
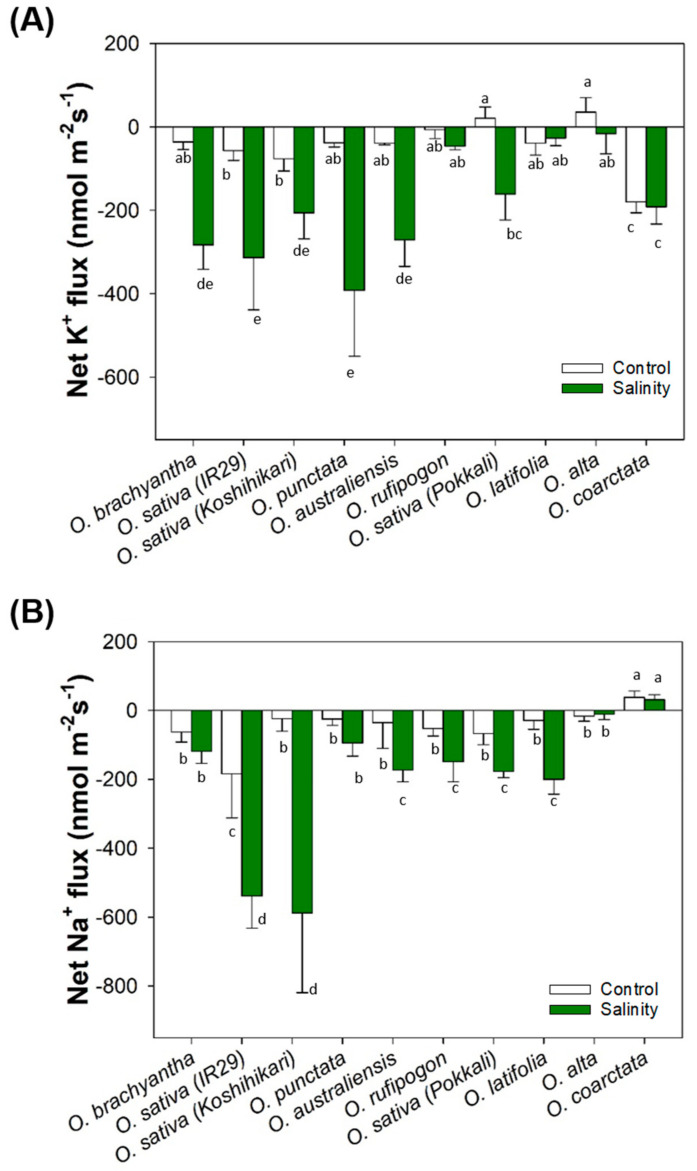
Effects of salinity on steady-state net ion fluxes of leaf mesophyll after 4 weeks of salinity stress. Data are net K^+^ (**A**) and Na^+^ (**B**) fluxes from leaf mesophyll collected from control and salinity-stressed plants. Different lowercase letters indicate significant differences at *p* = 0.05 (n = 4–8 biological replicates) according to Duncan’s Multiple Range Test.

**Figure 8:**
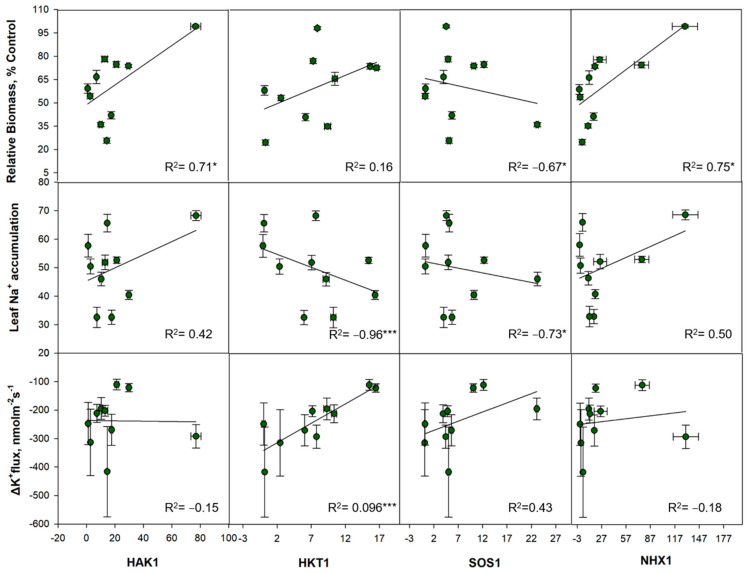
Relationship between phenotypic response to salinity stress and gene expression of salinity ion transporters. Pearson correlations among phenotypic factors; biomass, leaf Na^+^ accumulation and shift in K^+^ fluxes by pairwise comparison to relative gene expression under salinity stress of *HAK1*, *HKT1*, *SOS1*, and NHX, *** and * indicate significant correlation at *p* < 0.001 and *p* < 0.05, respectively.

To validate this data, the steady state fluxes of Na^+^ and K^+^ were also measured from leaf mesophyll cells of wild and cultivated rice species. Compared to the sensitive lines (*O. brachayantha*, *O. sativa cv*. IR29, and *O. sativa cv*. Koshihikari), most wild rice species with moderate to high salinity tolerance demonstrated relatively lower Na^+^ effluxes. Halophytic *O. coarctata* even showed a small Na^+^ influx in both control and salt-treated leaves (Figure 7A). In addition, steady state K^+^ fluxes from leaf mesophyll cells were not affected by salinity treatment in the highly tolerant species (*Pokkali*, *O. latifolia*, *O. alta*, and *O. coarctata*), while the rest of the *Oryza* species examined exhibited significant NaCl induced K^+^ effluxes (Figure 7B). Correlation analysis between gene expression and the physiological traits measured showed that upregulation of genes related to tissue tolerance (*NHX1* and *VHA-c*) coupled with upregulation of *HAK1* contributed to lower biomass reduction in the salt-tolerant wild rice species. It also showed that Na^+^ exclusion from leaf mesophyll cells is highly correlated with upregulation of *HKT1* and *SOS1* (Figure 8 and Figure S6).

## 3. Discussion

### 3.1. Molecular and Evolutionary Implications of Rice NHXs for Salinity Tolerance

The *NHX* gene family has been widely reported as a potential target for engineering resistance in plants against abiotic stress, especially salinity stress [36,37,39,43,47,48,49,50]. Plant NHXs are important transporters that mediate the coupled exchange of Na^+^ and K^+^ for H^+^ in all cellular compartments [37]. NHX transporters belong to the CPA superfamily, and CPA1 proteins [51,52,53] can be sub-grouped based on their cellular location (Figure 2A). NHXs are grouped into three classes: plasma membrane-, vacuolar-, or endosomal-NHXs. NHXs within each class showed high similarity and are present in a diverse range of plants ranging from streptophyte algae to angiosperms, suggesting location-specific evolution of these transporters [54]. This finding also supports the notion that NHX transporters were present in ancestral algae before terrestrial colonization by land plants (Table S2) [49,53].

In rice, in response to salinity stress, there are two well-characterized NHX transporters: plasma membrane Na^+^/H^+^ transporter SOS1 (also known as NHX7) and tonoplast Na^+^/H^+^ antiporter NHX1. SOS1 facilitates Na^+^ efflux from the cell to apoplast [55,56,57,58], while NHX is involved Na^+^ sequestration in vacuoles [37]. Both these proteins have the same Na^+^/H^+^ exchanger domain but differ significantly from each other at the C-terminus (Figure 2, [49]). Molecular evolutionary analysis by Pires et al. [52] reported distinct and independent evolutionary histories for these two NHX transporters. SOS1 showed constrained purifying selection, leading to few gene copies among green plant species compared to a greater number of vNHXs found from algae to land plants (Table S2) [52,59]. Genome-wide in silico identification and characterization of NHXs also reported a high copy number of vac-NHXs genes (12 out of 16) in indica rice [60]. Here, we show that four out of the seven putative NHX proteins belong to the vacuolar class of NHX transporters (Figure 2) identified in the cultivated rice Nipponbare genome [36]. These data suggest that vacuoles and tonoplast transporters, such as vNHXs, play important roles in plant life and that they use this cellular compartment and the high tonoplast transport capacity to constantly adjust to harsh environments, such as salinity [61,62].

Keeping this in mind, among the reported vacuolar class NHXs found in rice, *OsNHX1* is the most abundant, and its upregulation is highly induced by salt, drought, and ABA. Overexpression of *OsNHX1* showed higher tolerance of transgenic plants [48,63]. Here, we have compared NHX1 organization and structure across the available *Oryza* genomes to examine its potential contribution to salinity tolerance in *Oryza* species. Phylogenetic analysis showed that the origin of the gene in the halophytic wild rice, *O. coarctata*, was basal, suggesting the early divergence of this species within the genus *Oryza*. In the course of evolution, this species may have evolved earlier compared to other wild *Oryza* relatives (Figure 1A and Figure 3A). The natural habitat of *O. coarctata* also suggests that the evolution of many salinity-responsive genes may have been lost in domesticated rice during this transition from saline to fresh water [64]. Interestingly, although cultivated rice is taxonomically related to its halophytic wild rice relative to *O. coarctata*, NHX1 in cultivated rice *O. sativa* is more closely related to NHX1 homologues in glycophytic grasses than to *O. coarctata* (Figure 1A). In addition, a proteomics study showed that less than 50% of the differentially expressed proteins related to halophilic physiology of *O. coarctata*, such as those related to high photosynthetic capacity, are found in cultivated rice [65]. This also suggests that genetic variation for high salinity tolerance may have been lost during the course of domestication [66,67], which is consistent with evidence found in other species. This is similar to work on cultivated wheat that lacks the sodium transporters *Nax1* and *Nax2*, which are found in ancestral wild wheat relatives [68]. Additionally, a large natural variation among *SlHAK20* sequences was associated with differential salinity tolerance between wild and domesticated tomato species [69]. Future experiments should focus on the dissection of the 3D structures, key domains, and point mutations of NHX1s in both halophytic and glycophytic crops. These data may lead to the identification of genes that confer greater abilities to sequester Na^+^ into vacuoles leading to improved salinity tolerance in rice and other salt-sensitive crops.

### 3.2. Tissue Tolerance via Na^+^ Sequestration for Salt Tolerance in Wild Rice

When plants are in a saline environment, they can adapt by restricting Na^+^ influx into the cells. However, due to its strong driving force for entry, the ratio of influx vs. efflux rates will eventually cause cells to accumulate Na^+^ initially in the cytosol of root cells and then in other plant tissues [37,70,71]. Additionally, Na^+^ exclusion processes are highly energy driven and can further increase the ionic and osmotic imbalance in the cell [72,73,74]. The Na^+^ exclusion strategy is only useful in the short term and cannot counter the long-term effect of salinity stress in natural environments [75]. Therefore, tissue tolerance is likely to be a better option for plants to survive long-term salinity stress, where Na^+^ ions are sequestered in the vacuole to allow cellular functions and processes to continue unhindered [25]. Tissue tolerance is also important for osmotic adjustment, as it eases the Na^+^ load in the cytosol and allows the growth and expansion of cells with Na^+^ in vacuoles serving as a cheap osmoticum [76]. This is also a defining contrasting trait between halophytes and glycophytes. Halophytes accumulate substantial concentrations of Na^+^ in leaf tissues and maintain optimal K^+^/Na^+^ ratio by Na^+^ sequestration into vacuoles [28,77,78]. In our study, the salt-tolerant and halophytic wild rice species accumulated relatively high concentrations of Na^+^ in their leaves (Figure 5), implying the existence of a Na^+^ vacuolar sequestration process. Vacuolar Na^+^ sequestration in the leaf mesophyll of wild rice is mediated by *NHX1* and the proton pump, *VHA-c*, which is highly upregulated upon exposure to salinity stress (Figure 6A). The upregulation of VHA pump activity is required to drive vacuolar Na^+^ sequestration, and this allows the transport of metabolites and ions between cytosolic and vacuolar pools depending on the metabolic demand of salinity stressed plants [79]. A similar tissue tolerance mechanism was observed in an extremophile shrub, *Zygophyllum xanthoxylum*, where the accumulation of Na^+^ ions in the leaves resulted in mesophyll enlargement and leaf succulence, preventing water loss [80], and in a woody halophyte, *Nitraria sibirica*, where the excessive Na^+^ sequestration in vacuoles in the shoots correlated with upregulation of *NsVHA*, *NsVP1* and *NsNHX1* transcripts [81].

In contrast, upregulation of K^+^ retention/Na^+^ exclusion genes (*SOS1/NHX7*, *HAK1*, *HKT*) was more evident in the salt-tolerant cultivated rice, Pokkali. In addition, these ion transporters are also significantly upregulated in wild rice species and expressed at even higher levels compared to Pokkali, most notably *HAK1* [78,79,80]. This suggests that wild rice could effectively balance both Na^+^ exclusion and inclusion governed by *NHX1, SOS1*, and *HAK1* to avoid the high energy-driven process of total Na^+^ exclusion [70,81]. Moreover, excessive accumulation of toxic Na^+^ ions in the shoot will lead to leaf senescence. However, in this study, the wild rice species did not show significant growth and biomass reductions even in the presence of relatively large amounts of Na^+^ ions in the leaf. This may indicate that salt-tolerant wild rice species efficiently use the Na^+^ accumulated as cheap osmoticum by sequestering Na^+^ to mesophyll vacuoles, compared to high energy driven Na^+^ exclusion processes with concomitant less yield penalty [82].

### 3.3. Divergent Evolution of Halophytic NHXs for Developing Salinity Tolerant Rice

A key question in evolutionary studies is how species in diverse lineages acquire the same trait, such as salinity tolerance, a complex trait that has evolved multiple times in land plants [83]. Thus, understanding the nature and evolution of salinity tolerance will facilitate the development of salinity-tolerant crops. Wild rice species possess useful traits that can potentially improve plant performance under salinity stress. Efforts have been made to identify salinity-tolerant genetic resources for breeding salinity-tolerant rice. However, most work has been focused on Na^+^ exclusion, while traits, such as tissue tolerance, have received less attention. Previous reports have shown that wild rice species exhibit superior tolerance of higher Na^+^ concentrations in their tissues; however, details regarding the underlying mechanisms remain elusive [22,23]. In this study, we present evolutionary and functional evidence that tissue tolerance in wild *Oryza* species is mediated by vacuolar Na^+^ sequestration driven by *NHX1*.

Salinity tolerance has evolved in lineages of closely related crops and is likely associated with geographical and environmental adaptation [84]. The diversity of the genus *Oryza* would suggest some key salinity tolerance properties that can be used in rice salinity breeding platforms. The wild relatives of *Oryza sativa*, such as *O. latifolia, O. glaberrima*, and *O. coarctata* that grow in aquatic marshes, possess unique morphological salt adaptive features, such as waxy leaves, the presence of rhizomes and ligules, and even features of C_4_ photosynthesis. This indicates that members of the genus *Oryza* may have undergone significant differentiation during evolution [85,86]. During the course of domestication and continuous artificial selection during plant breeding, genetic diversity in *Oryza sativa* has been significantly reduced in many modern rice cultivars [87], and only 10–20% genetic diversity from wild rice has been retained in cultivated rice [88]. The impact of these genetic changes have caused the loss of beneficial traits, such as tolerance to salinity, through gene loss, mutation, and structural variation, but there is the potential to restore these traits in modern crops. NHX1 in *Suaeda salsa* and SOS1 in *Salicornia brachiata*, both of which are halophytes, conferred salinity tolerance when these genes were overexpressed in salinity susceptible plant species, such as *Arabidopsis* and tobacco [89,90]. In *Oryza*, although OsNHX1 and OcNHX1 share high identity, differential expression patterns were observed (Figure 3 and Figure 6). This variation in amino acid sequence could potentially result in higher tolerance in *O. coarctata.* As reported in the complementation studies using salt-sensitive yeast strains [30], OcNHX1 confers better growth under increasing saline conditions compared to that conferred by OsjNHX1 from *O. sativa subsp. japonica* and OsiNHX1 from *O. sativa subsp. indica*. A similar pattern was observed by the upregulation of *vNHXs* in a halophyte *Helianthus tuberosus* [91] and in wheat *Ta*NHX1 [50]. An understanding of the existence and magnitude of such diversification in *NHXs*, especially in the genus *Oryza*, could be highly valuable in finding sources of germplasm to assist breeding of salinity tolerant rice.

## 4. Materials and Methods

### 4.1. Evolutionary Bioinformatics

Evolutionary bioinformatics were conducted following Zhao et al. [92] and Feng et al. [93]. Putative NHX genes in the genome of *Oryza sativa* were retrieved from the Rice Genome Annotation Project database (http://rice.plantbiology.msu.edu/ accessed on 1 November 2021) [94]. Using Arabidopsis NHXs as query sequences, protein sequences with homology to vacuolar NHX1 were mined from the 1000 Plant Transcriptome (1KP) [95] and Phytozome (V13) (https://phytozome-next.jgi.doe.gov/ accessed on 1 November 2021) databases [96]. Alignment of protein sequences was obtained using MUSCLE [97] and was defined using the Gblocks default setting [98]. The best-fit amino acid substitution model was selected using ProTest from MEGAX [99]. The conserved domains of all candidate NHX amino acid sequences were identified using the Simple Modular Architecture Research Tool (SMART) [100] Phylogenetic trees were constructed with FastTree using maximum likelihood with 1000 bootstrap replicates; the Interactive Tree of Life resource (https://itol.embl.de/ accessed on 1 November 2021) was used to annotate and visualize the phylogenetic trees [101].

MEME software (Multiple Expectation Maximization for Motif Elicitation; Version 5.4.1) (https://meme-suite.org/meme/tools/meme/ accessed on 1 November 2021) was used to identify conserved motifs and to describe their position and frequency [102]. The coding sequences and corresponding genomic DNA sequences were downloaded as described in [94], and Gene Structure Display Server 2.0 (http://gsds.gao-lab.org/ accessed on 1 November 2021), a web-based bioinformatics tool, was used to visualize gene features concerning intron-exon organization of rice NHX genes. The transmembrane helix prediction and hydrophobic regions of representative NHX proteins were analyzed with the topology prediction web-based tool PROTTER [103]. The conserved domains in each protein sequence were identified by motif scan using SMART (http://smart.embl-heidelberg.de/smart/ accessed on 1 November 2021) [100] and InterPro (http://www.ebi.ac.uk/interpro/ accessed 1 November 2021) [104], and 3D structures were predicted using SWISS-MODEL (https://swissmodel.expasy.org/ accessed 1 November 2021) [105].

### 4.2. Plant Materials and Salinity Treatment in a Field Trial

Nine wild *Oryza* species representing the primary (*O. rufipogon*), secondary (*O. punctata*, *O. alta*, *O. latifolia*, *O. australiensis*), and tertiary (*O. brachyantha, O. coarctata*) gene pools together with the cultivated rice (*O. sativa*) salinity tolerant line, Pokkali, and salinity sensitive lines, IR29 and Koshikari, were grown under prolonged high salinity stress (EC = 12 dS m^−1^) and compared to controls (EC = 0.5 dS m^−1^) in a field trial. Salinity stress was imposed at the early vegetative stage, leaf tissues were collected after the 4th week of salinity treatment, and visual symptoms of stress were recorded. Leaves were also harvested for element analysis, ion flux measurement, confocal imaging, and qPCR experiments. Shoots were harvested to determine biomass and shoot length. Mean values were obtained from 12 independent biological replicates (1 m^2^) per block.

### 4.3. Ion Flux Measurement

Steady-state net K^+^ and Na^+^ fluxes were measured from leaf mesophyll cells of flag leaves 1 h after recovery from excision using non-invasive microelectrode ion flux estimation (MIFE) conducted according to Shabala et al. [106] with the following modifications. Prior to the measurement, cross-sectioned leaf samples were clamped in a Perspex measuring chamber and submerged in standard MIFE solution (0.5 mM KCl, 0.1 mM CaCl_2_) for 1 h. The measurements were performed using 8–12 biological replicates for each cultivar after the 4th week of salinity stress. Steady-state ion flux readings were taken for 10 min for each sample. Net ion fluxes were calculated using MIFEFLUX software (designed, manufactured and distributed by the University of Tasmania, http://www.phys.utas.edu.au/physics/biophys/mifecom/MIFESoftware/Software.htm/ accessed on 14 January 2022) based on the ion concentration gradient recorded between two-point positions.

### 4.4. Confocal Microscopy

Na^+^ accumulation in leaf mesophyll cells of freshly harvested flag leaves was measured using confocal microscopy following Wang et al. [107] and Wu et al. [108]. The epidermal layer of the flag leaves was removed from sections of leaf prior to incubation of the sections in buffer (10 mM KCl, 5 mM Ca^2+^-MES, pH 6.1) containing the appropriate dye. CoroNa Green acetoxymethyl (AM) ester was used to measure the relative Na^+^ ions accumulated in leaf mesophyll cells. The stained tissues were washed in distilled water for 3 min to remove unincorporated dye before imaging. Measurements were performed on two representative areas of each section using an upright laser scanning confocal microscope (Leica, Göttingen, Germany) fitted with a 50× objective (laser power: 10%, excitation wavelength: 488 nm, emission range: 505–550 nm, filter: TP488/543/633). Na^+^ florescence intensities were then quantified on the images taken on a cell-by-cell basis.

### 4.5. Leaf Tissue Na^+^ Contents

Shoots were harvested at full maturity after exposure to prolonged salinity stress from the early vegetative stage. Two to three leaves were collected and oven dried for 3 days at 65 °C. Approximately 50 mg of dried leaf samples were finely ground and mixed using a mortar and pestle, and tissue digestion was performed using 4 mL of concentrated HNO_3_ (69%) in a boiling water bath until the sample solution was clear. The tissue extracts were filtered using Whatman filter paper and diluted with milliQ water. The Na^+^ concentration was measured in percentage (% g^−1^ dry weight) using a flame photometer (Jenway PFP7, John Morris, Sydney, Australia).

### 4.6. Quantitative Real Time PCR

Quantitative real time PCR was conducted according to Liu et al. [109] to measure the gene expression of key transporters related to salt tolerance in rice. Total RNA from rice leaves was extracted in the 4th week after stress application using TRIzol reagent (Invitrogen, Carlsbad, CA, USA), and reverse transcription was performed using a SensiFAST cDNA synthesis kit (Bioline, London, UK) according to the manufacturer’s instructions. The expression of genes (*HAK1, NHX1*, *HKT1;4*, *SOS1*, and *VHA-c*) known to be involved with salt tolerance was assessed by real-time quantitative RT-PCR using a Quantinova Sybr Green Kit (Qiagen, Valencia, CA, USA) in a Rotor-Gene 3000 quantitative PCR thermocycler (Corbett Research, Mortlake, NSW, Australia). *G6PDH* and *Elfa* were used as reference genes.

### 4.7. Data Analysis

The SAS/STAT^®^ 9.4 (SAS Institute Inc., Cary, NC, USA) was used to perform two/three-way analyses of variance (ANOVA), followed by Duncan’s Multiple Range Tests (DMRT); an alpha = 0.05 or less was considered significant. For all the relative value calculations, the average data in the salinity treatment was divided by the average of the control. The species used in the study were ranked based on their overall performance under salinity stress. The relative value of the parameters measured (Table S6) from the control and salinity treatment was calculated to evaluate the salinity sensitivity of the different *Oryza* species. DMRT was used to rank the relative value of each parameter that was listed in Table S6, using the ANOVA-post hoc tool in SAS/STAT^®^ software. The performance of *O. sativa* tolerance checks, such as Pokalli (tolerant check) and IR29 and Koshikari (susceptible check), was used as indicators for ranking of homogenous groups. The ranking of the homogenous groups of each parameter started from 1 as the most sensitive group and increased based on the numbers of homogenous groups from the DMRT result. This ranking was used in the arrangement of species for data presentation. All graphs and tables were prepared using SigmaPlot 12.0 (Systat Software, Inc., San Jose, CA, USA) and Office 365 Excel (Microsoft Corporation, Redmond, WA, USA).

## 5. Conclusions

In the present study, we evaluated salinity tolerance in accessions of wild and cultivated rice. Comparative sequence analysis of NHX transporters coupled with gene expression assays provided insight into the diversification and evolution of tissue tolerance in *Oryza* species. In rice, even though *O. sativa* and *O. coarctata* are taxonomically related and the sequences are highly conserved, sufficient differences in the sequences of these NHX genes cause the homologue in *O. coarctata* to cluster with homologues from other halophytic grasses. Here, the discovery of evolutionary divergence of NHXs and their association with tissue tolerance, a key trait observed in wild *Oryza* species, could be useful to facilitate the identification of novel resources of useful germplasm to increase genetic variation for salt-tolerant rice breeding. NHX is highly expressed regardless of salinity tolerance in wild rice.

## Data Availability

The data are available from the authors upon request.

## Supplementary Materials

The following supporting information can be downloaded at: https://www.mdpi.com/article/10.3390/ijms23042092/s1.
