# Supplementary material for: Evolutionary Significance of NHX Family and NHX1 in Salinity Stress Adaptation in the Genus Oryza
Source: Int J Mol Sci. 2022 Feb 14;23(4):2092. doi: 10.3390/ijms23042092 (PMC8879705; doi:10.3390/ijms23042092)
Supplement: Supplementary file 1 [file ijms-23-02092-s001.zip › ijms-1510054-supplementary.pdf]

Species	*Arabidopsis thaliana*	*Oryza sativa*	*Oryza coarctata*	*Zea mays*	*Picea sitchensis*	*Pteris vittata*	*Selaginella moellendorffii*	*Marchantia polymorpha*	*Klebsormidium nitens*	*Volvox carteri*	*Dictyostelium discoideum*
Arabidopsis thaliana		80.6	83.1	78.4	82.3	84.1	80.7	84.1	78.4	67.4	59.4
Oryza sativa	80.6		88.6	84.8	88	83.8	81.9	83.6	80	68.1	59.7
Oryza coarctata	83.1	88.6		86.2	90.2	86.9	83	85.1	79.5	66.6	62
Zea mays	78.4	84.8	86.2		84.4	83.3	76.3	78.9	76.9	65.6	60.3
Picea sitchensis	82.3	88	90.2	84.4		87.2	85	86.2	79.6	66.6	60.9
Pteris vittata	84.1	83.8	86.9	83.3	87.2		85.8	90	82.5	64.8	64.6
Selaginella moellendorffii	80.7	81.9	83	76.3	85	85.8		84.4	78.4	66.4	61
Marchantia polymorpha	84.1	83.6	85.1	78.9	86.2	90	84.4		82.1	63.9	61.3
Klebsormidium nitens	78.4	80	79.5	76.9	79.6	82.5	78.4	82.1		66.5	56.3
Volvox carteri	67.4	68.1	66.6	65.6	66.6	64.8	66.4	63.9	66.5		57.2
Dictyostelium discoideum	59.4	59.7	62	60.3	60.9	64.6	61	61.3	56.3	57.2	

Figure S1. Sequence identity of *NHX1* in representative plant species.

	OsNHX1	*OsNHX2*	*OsNHX3*	*OsNHX4*	*OsNHX5*	*OsNHX6*	*OsNHX7*
OsNHX1		81.7	80.3	63	28.4	35	20.3
OsNHX2	81.7		79.9	62.6	30.1	34.5	20.6
OsNHX3	80.3	79.9		61.9	29.1	36.1	19.4
OsNHX4	63	62.6	61.9		26.8	29.8	18.1
OsNHX5	28.4	30.1	29.1	26.8		49.4	15.5
OsNHX6	35	34.5	36.1	29.8	49.4		14.9
OsNHX7	20.3	20.6	19.4	18.1	15.5	14.9	

Figure S2. Sequence identity of putative NHXs mapped in *Oryza sativa.*

	O. sativa subsp. japonica	*O. sativa subsp. indica*	*O. glaberrima*	*O. nivara*	*O. rufipogon*	*O. meridionalis*	*O. glumaepatula*	*O. punctata*	*O. barthii*	*O. brachyantha*	*O. coarctata*
O. sativa subsp. japonica		91.8	91.8	91.6	91.4	91.4	87.9	90.3	90.6	89	89.3
O. sativa subsp. indica	91.8		100	99.8	99.6	99.6	95.7	98.5	99.6	97.9	97.2
O. glaberrima	91.8	100		99.8	99.6	99.6	95.7	98.5	99.6	97.9	97.2
O. nivara	91.6	99.8	99.8		99.8	99.4	95.9	98.3	99.4	98.1	96.8
O. rufipogon	91.4	99.6	99.6	99.8		99.3	95.7	98.1	99.2	97.9	96.8
O. meridionalis	91.4	99.6	99.6	99.4	99.3		95.3	98.7	99.2	98.1	97.2
O. glumaepatula	87.9	95.7	95.7	95.9	95.7	95.3		94.2	95.2	93.5	92.9
O. punctata	90.3	98.5	98.5	98.3	98.1	98.7	94.2		98.3	98.3	97.8
O. barthii	90.6	99.6	99.6	99.4	99.2	99.2	95.2	98.3		97.5	97.5
O. brachyantha	89	97.9	97.9	98.1	97.9	98.1	93.5	98.3	97.5		97.9
O. coarctata	89.3	97.2	97.2	96.8	96.8	97.2	92.9	97.8	97.5	97.9	

Figure S3. Sequence identity of NHX homologues identified in the *Oryza* genus.

	Logo	E-value	Site	Width	Role
1.	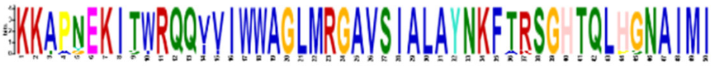	1.4e-514	12	50	
2.	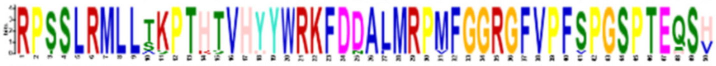	3.2e-495	12	50	
3.	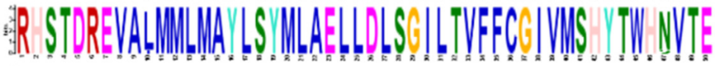	1.0e-494	12	50	
4.	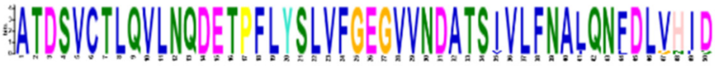	2.5e-478	12	50	
5.	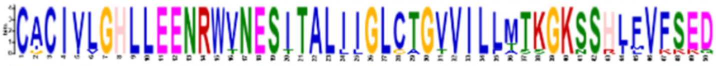	2.4e-337	10	50	
6.	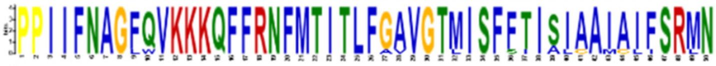	2.7e-339	10	50	
7.	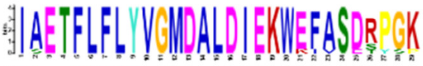	3.0e-229	11	29	Aspartate-alanine exchange (Asp-Al_Ex)
8.	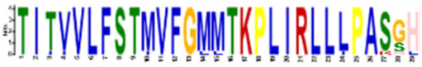	1.2e-217	12	29	
9.	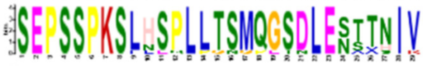	1.9e-216	12	29	Monovalent Cation:Proton Antiporter-1 (CPA1)
10.	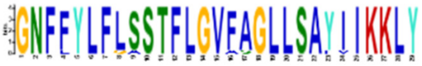	2.9e-215	12	29	

Figure S4. Various protein motifs found in representative *Oryza* NHX1s.

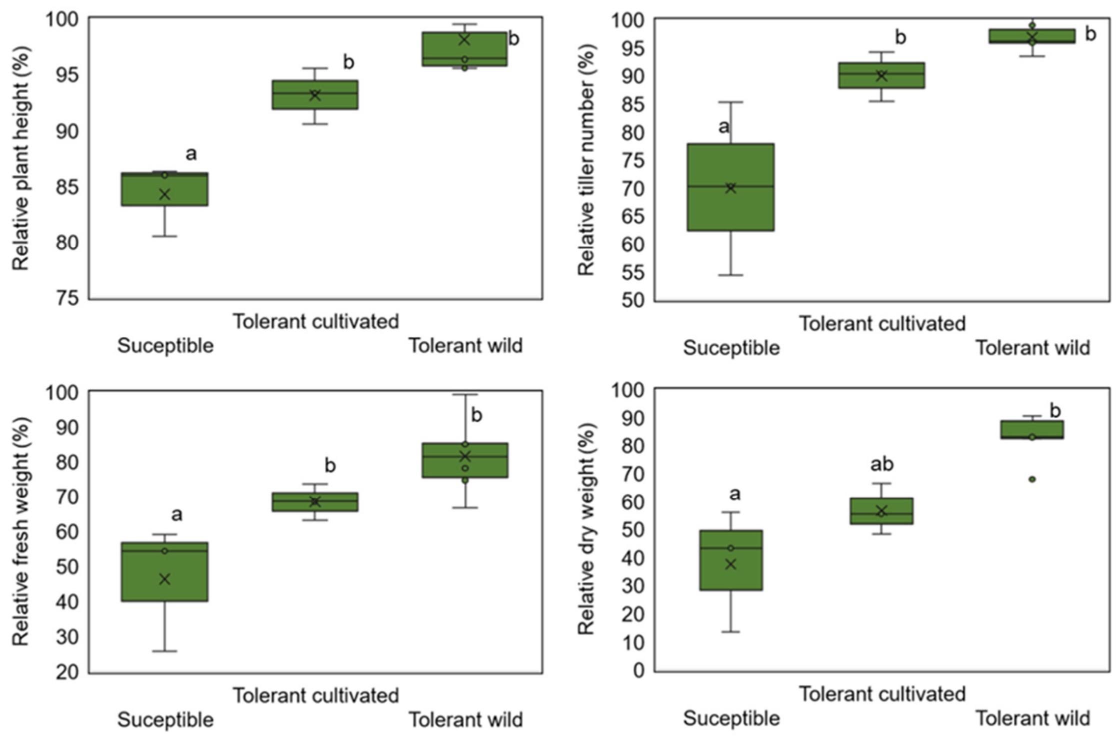

Figure S5. Variation of salinity tolerance in Oryza species. Phenotypic variation on the effect of salinity treatment on overall growth, Relative effects (% of control) of salinity on plant height, tiller number, fresh weight, and and dry weight. Different lowercase letters indicate significant differences at $P < 0.05$ ($n = 3–6$ Oryza lines) according to Duncan's Multiple Range Test.

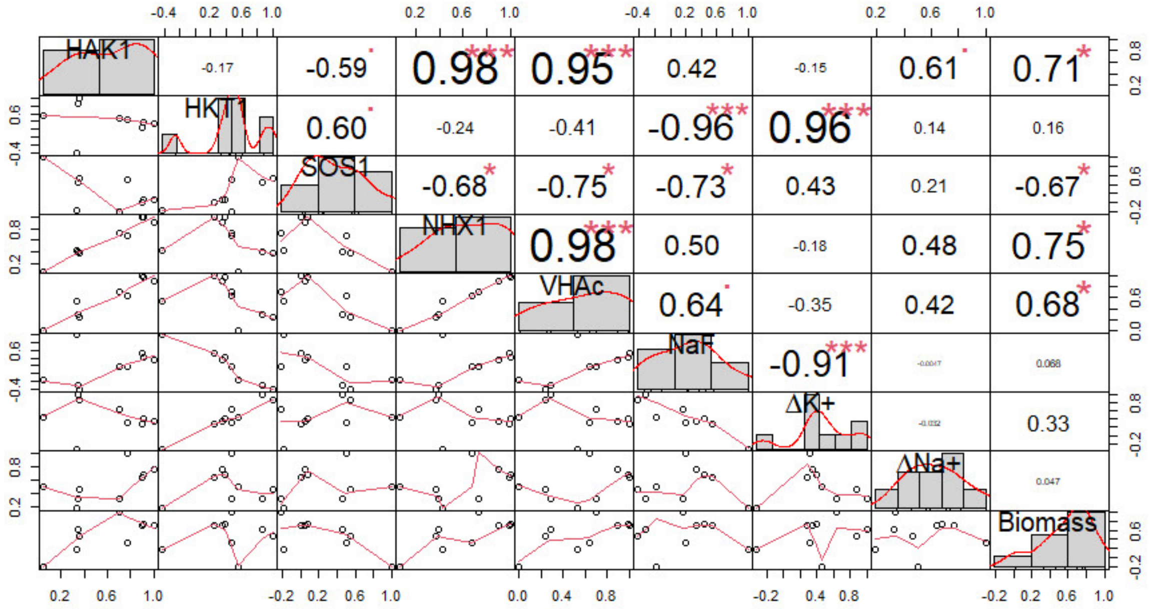

Figure S6. Relationship of gene expression of salinity transporters in ion transport and yield in response to salinity stress in cultivated and wild rice species. Spearman correlation matrix and histogram of phenotypic traits observed and gene expression of known salinity tolerant genes. The distribution of each variable is shown on the diagonal. ***, **, * indicate significant correlation at $P<0.001$, $P<0.01$ and $P<0.05$. NaF- relative mesophyll Na^+ fluorescence, ΔK^+- K^+ flux difference(stress-control), ΔNa^+- Na^+ flux difference (stress-control), Biomass – relative above ground biomass harvested at full maturity, *HAK1, HKT1, SOS1* , *NHX1* and *VHAc* - relative gene expression under salinity stress.

Table S1. Identification and sequence analysis of NHX1 in representative species.

Classification	Species	Length (aa)	MW (kD)	PI
Dicot	*Arabidopsis thaliana*	529	58.87	7.84
	Oryza sativa	545	59.91	7.93
Monocot	*Oryza coarctata*	539	59.45	7.85
	Zea mays	515	57.55	8.21
Gymnosperm	*Picea sitchensis*	535	59.09	8.29
Fern	*Pteris vittata*	463	50.91	6.1
Lycophyte	*Selaginella moellendorffii*	532	58.93	8.58
Liverwort	*Marchantia polymorpha*	521	57.64	6.62
Steptophyte Alga	*Klebsormidium nitens*	547	59.69	6.65
Chlorophyte Alga	*Volvox carteri*	474	50.95	9.56
Dictyosteliida	*Dictyostelium discoideum*	674	75.52	4.31

Table S2. Number of NHX transporters mapped in representative species.

		NHX subtypes			
Classifications	Species	Vac	PM	TGN	Total
Algae	*Chlamydomonas reinhardtii*	1	3	1	5
Bryophytes	*Physcomitrella patens*	5	2	2	9
Lycophytes	*Selaginella moellendorffii*	2	2	2	6
Gymnosperm	*Picea sitchensis*	1	?	?	?
Monocots	*Zea Mays*	6	2	2	10
	Sorghum bicolor	4	2	1	7
	Oryza sativa	4	2	1	7
Eudicots	*Arabidopsis thaliana*	4	4	2	10
	Gossypium hirsutum	11	23	17	1
	Solanum lycopersocum	5	3	1	9
	Glycine max	11	7	3	21

Table S3. The identification and sequence analysis of the Na^+/H^+ antiporter (NHX) gene family in rice.

Gene	Rice MSU Locus ID	Length (aa)	MW (kD)	PI	Subcellular Localization
OsNHX1	LOC_Os07g47100.2	536	59.07	8.3	Vacoular
OsNHX2	LOC_Os11g42790.1	546	59.92	7.9	Vacoular
OsNHX3	LOC_Os05g05590.1	529	59.64	7.3	Vacoular
OsNHX4	LOC_Os06g21360.1	560	58.22	6.3	Vacoular
OsNHX5	LOC_Os09g11450.1	550	60.39	5.7	Plasma Membrane
OsNHX6	LOC_Os09g30446.1	462	31.07	5.5	Plasma Membrane
OsNHX7	LOC_Os12g44360.2	1149	127.91	6.8	Trans-Golgi Network

Table S4. Number of NHX transporters mapped in in representative *Oryza* species.

Species	Vac Class	Gene hit	PM Class	Gene hit	TGN Class	Gene hit
Oryza sativa subsp. indica	4	BGIOSGA027380<br>BGIOSGA026347<br>BGIOSGA018801<br>BGIOSGA029567	1	BGIOSGA035749	2	BGIOSGA029567<br>BGIOSGA030066
Oryza sativa subsp. japonica	4	OsNHX1<br>OsNHX2<br>OsNHX3<br>OsNHX4	1	SOS1	2	OsSTA230<br>OsNHX5
Oryza rufipogon	4	ORUFI05G03390<br>ORUFI06G13400<br>ORUFI07G26490<br>ORUFI11G23040	1	ORUFI09G04390	2	ORUFI09G15320<br>ORUFI12G22630
Oryza meridionalis	3	OMERI05G03260<br>OMERI07G22210<br>OMERI11G17560	0	?	2	OMERI09G03010<br>OMERI09G11060
Oryza nivara	4	ONIVA05G03230<br>ONIVA06G14580<br>ONIVA07G25170<br>ONIVA11G21550	1	ONIVA12G18960	2	ONIVA09G04150<br>ONIVA09G14830
Oryza glaberrima	4	ORGLA09G0109500<br>ORGLA07G0266400<br>ORGLA07G0198900<br>ORGLA05G0031000	1	ORGLA12G0176700	2	ORGLA09G0109500<br>ORGLA12G0176700
Oryza barthii	4	OBART05G03250<br>OBART06G12670<br>OBART07G25570<br>OBART11G21190	1	OBART12G20310	2	OBART09G03930<br>OBART09G14270
Oryza longistaminata	4	AMDW01040861.1_<br>F<br>KN538696.1_FG089<br>KN539398.1_FG001<br>KN540366.1_FG005	1	KN538756.1_FG016	2	KN539227.1_FG004<br>KN538880.1_FG014
Oryza glumipatula	4	OGLUM05G03300<br>OGLUM06G13560<br>OGLUM07G25540<br>OGLUM11G20600	1	OGLUM12G22080	2	OGLUM09G04670<br>OGLUM09G14920
Oryza punctata	4	OPUNC05G03110<br>OPUNC06G12060<br>OPUNC07G23980<br>OPUNC11G17770	1	OPUNC12G18600	2	OPUNC09G12770
Oryza brachyantha	5	OB05G13170<br>OB06G21140<br>OB07G30930<br>OB11G25980<br>OB12G17900	2	OB09G20810<br>OB12G27070	5	OB0092G10010<br>OB04G14430<br>OB09G12460<br>OB09G12470<br>OB12G18280

Table S5. Identification and sequence analysis of NHX1 in representative *Oryza* species.

NHX1 species	Genome	Length (aa)	MW (kD)	PI
O. sativa subsp. japonica	AA	535	59.07	7.77
O. sativa subsp. indica	AA	498	54.85	8.34
O. glaberrima	AA	535	59.07	8.34
O. nivara	AA	535	59.07	8.34
O. rufipogon	AA	535	59.07	8.34
O. meridionalis	AA	536	59.16	8.34
O. glumaepatula	AA	513	56.52	8.84
O. longistaminata	AA	539	59.88	9.51
O. barthii	BB	475	52.70	9.56
O. punctata	BB	536	58.95	8.10
O. brachyantha	FF	477	52.74	9.27
O. coarctata	KKLL	539	59.45	7.85

Table S6. Salinity ranking of *Oryza* species based on the relative value of the physiological parameters.

Species	Genome	Gene Pool	RFW	RDW	RWC	Na^+ accumulation	ΔK^+	ΔNa^+	Overall	Tolerance Rank
O. brachyantha	FF	Tertiary	1	1	3	1	1	1	1.2	Susceptible
O. sativa cv. IR29	AA	Primary	2	3	4	2	2	2	3.4	Susceptible
O. sativa cv. Koshihikari	AA	Primary	3	2	7	3	3	3	3.6	Susceptible
O. punctata	BB	Secondary	4	4	1	5	4	5	3.9	Moderately Tolerant
O. rufipogon	AA	Secondary	5	5	2	4	5	4	3.9	Moderately Tolerant
O. australiensis	EE	Secondary	6	6	6	6	6	6	6	Moderately Tolerant
O. sativa cv Pokkali	AA	Primary	7	7	5	7	7	7	7.2	Tolerant
O. latifolia	CCDD	Secondary	8	8	9	8	8	8	7.4	Tolerant
O. alta	CCDD	Secondary	9	9	8	9	9	9	8.6	Tolerant
O. coarctata	KKLL	Tertiary	10	10	10	10	10	10	10	Highly Tolerant

Abbreviations: RFW- relative fresh weight, RDW- relative dry weight, RWC- relative water content, Na^+ accumulation- relative mesophyll Na^+ fluorescence, ΔK^+- K^+ flux difference(stress/control), ΔNa^+- Na^+ flux difference (stress/control).
